# Static Electro-Mechanical Response of Axisymmetric One-Dimensional Piezoelectric Quasicrystal Circular Actuator

**DOI:** 10.3390/ma15093157

**Published:** 2022-04-27

**Authors:** Linyan Zhang, Hongliang Zhang, Yang Li, Jingbo Wang, Changguo Lu

**Affiliations:** 1 College of Mechanical and Power Engineering, Yingkou Institute of Technology, Yingkou 115014, China; yklglinyan.zhang@outlook.com (L.Z.); yklgzhanghongliang@outlook.com (H.Z.); jbmail@126.com (J.W.); lucg301@126.com (C.L.); 2 Department of Aerospace Science and Technology, Space Engineering University, Beijing 101416, China

**Keywords:** piezoelectric quasicrystals, circular actuator, axisymmetric bending, state space method, exact solution

## Abstract

The piezoelectric effect of piezoelectric quasicrystalline materials is coexcited by phonon and phason fields. Piezoelectric quasicrystalline materials have excellent properties of both piezoelectric materials and quasicrystalline materials, which are expected to be used as actuators in the fields of aerospace, automotive, and intelligent manufacturing. Based on the three-dimensional elastic theory of piezoelectric quasicrystals, the state space equation for axisymmetric piezoelectric quasicrystal circular plate actuators is derived by using the state space method. Afterwards, the finite Hankel transformation is performed on the state equation, and a system of ordinary differential equations and corresponding boundary conditions are obtained. Finally, the exact solution of axisymmetric bending of one-dimensional hexagonal piezoelectric quasicrystal circular actuators under generalized elastic simply supported boundary conditions is obtained by using the propagator matrix method. Numerical results are given to compare the degradation results in this paper with those in the literature, and present the influences of the thickness-to-span ratio and stacking sequence on the phonon, phason, and electric fields when the surface of the laminated circular actuators is subjected to mechanical load. The exact solution obtained does not introduce any deformation assumption; therefore, the exact solution can provide references for numerical calculations of the mechanical behavior of piezoelectric quasicrystals.

## 1. Introduction

Piezoelectric materials, known as smart materials, are rapid response and high resolution, which make them increasingly popular as ideal candidates for actuators and sensors. Piezoelectric elements are usually incorporated with composite laminates to obtain better stiffness, lightness, and reliability [1], which are applied in aerospace, medical engineering, biotechnological engineering, micro-electromechanical systems, and other fields. In order to reasonably design the laminated piezoelectric devices, the precise deformation solution of such a structure must be well understood. Establishing an accurate analytical model is an effective means of predicting the deformation behavior of laminated piezoelectric devices. By means of the energy method, Wei and Xue [2] proposed a simple nonlinear model to study the bending wave in a piezoelectric laminated beam. A surface/interface piezoelectric theory was utilized by Zhu et al. [3] to model the nonlinear vibration control of sandwich nano-shells with functionally graded piezoelectric nanocomposite sensors and actuators. Based on the Runge–Kutta method, Dong et al. [4] presented the active vibration control of sandwich cylindrical shells with piezoelectric actuator/sensor layers. Dehsaraji et al. [5] used the modified couple stress theory to present a new three-dimensional framework for buckling analysis of functionally graded piezoelectric cylindrical nano/micro-shells. Min et al. [6] proposed an artificial neural network model to predict the displacement amplitude and natural frequency of piezoelectric actuated rectangular plates.

As a new solid configuration, quasicrystals (QCs) have long-range quasi-periodic translational order and rotational symmetry, yet they lack the three-dimensional periodicity and translational symmetry that was discovered by Shechtman et al. [7]. Different from the well-known phonon excitation, a new elementary excitation (phason) is also introduced to describe the rearrangements of atomic configurations in the elastic energy theory of QCs [8]. For piezoelectric QCs, it can be known that their piezoelectric effects are coexcited by phonon and phason fields [9]. Therefore, piezoelectric QCs may possess the superior characteristics of both QCs and piezoelectric materials, which are expected to be used as sensors and actuators [10]. Due to the unique properties and wide perspective of applications of piezoelectric QCs, a lot of research has been carried out. Fujiwara et al. [11] first presented the electronic structure and electron transport property of two-dimensional QCs. Due to the good symmetry of one-dimensional (1D) QCs, the piezoelectric effect of 1D QCs has received extensive attention from more scholars. By introducing two displacement functions and utilizing the rigorous operator theory, Li et al. [12] obtained a set of 3D general solutions to static problems of 1D hexagonal piezoelectric QCs. Zhou and Li [13] studied cracking problems in 1D hexagonal piezoelectric QCs and determined the exact closed-form phonon and phason stress and electric fields. Zhang et al. [14] applied the Legendre polynomial method to study the guided wave propagating in a 1D hexagonal piezoelectric QC plate. In terms of the complex function method, Li et al. [15] solved the problem of the interaction between a screw dislocation and an elliptical hole with two asymmetric cracks in a 1D hexagonal piezoelectric QC. By utilizing the pseudo-Stroh formalism, Li et al. [16] obtained an exact solution for a functionally graded multilayered 1D QC plate. Hu et al. [17] solved the problem of collinear interface cracks between 1D hexagonal piezoelectric QCs under anti-plane shear and in-plane electric loading. Based on the conformal mapping technique and analytical continuation theory, Hu et al. [18] investigated the circular cylindrical inclusions in an infinite 1D piezoelectric QC medium.

Axisymmetric circular piezoelectric actuators are a typical kind of smart device in engineering. As for axisymmetric problems, the governing equations reduce to ordinary differential equations, and thus the three-dimensional problem can be simplified. Such a simplification is not only mathematically convenient but also of practical implications, because many circular piezoelectric devices have axisymmetric characteristics in geometry, physics, and loading simultaneously. Therefore, axisymmetric piezoelectric problems have attracted a lot of attention from numerous scholars. In terms of the state space method, Ding et al. [19] made an investigation on the free axisymmetric vibration of transversely isotropic piezoelectric circular plates. By extending the state space method, Li et al. [20] studied the influence of the properties of functionally graded materials on piezoelectric quasicrystal circular plates. By using the direct displacement method, Wang et al. [21] studied the axisymmetric bending of transversely isotropic and functionally graded circular plates under arbitrarily transverse loads. Yang et al. [22] assumed the variable separation form of the displacement function and electrical potential function, and obtained the electro-elastic solution of the axisymmetric deformation problem of functionally graded piezoelectric circular plates. By utilizing the direct displacement method, Zhao et al. [23] studied the axisymmetric problem of a heterogeneous multiferroic circular plate subjected to electric loading. By virtue of the perturbation method, Lv et al. [24] studied the axisymmetric contact vibration of a rigid spherical punch on a piezoelectric half-space.

Due to the introduction of phason fields, the electro-elastic theory for piezoelectric materials cannot be directly applied to QCs. Therefore, it is necessary to develop some theories to predict the phonon–phason–electric coupling behaviors of piezoelectric QCs. To the best of the authors’ knowledge, research on the electro-elasticity of circular actuators made of homogeneous QCs has not yet been investigated. To this end, the axisymmetric circular piezoelectric quasicrystal plate model is established based on the state space method, which incorporates the phonon, phason, and electric fields simultaneously. By virtue of the finite Hankel integral transform, the state vector equations of axisymmetric piezoelectric QCs can be reduced to a system of ordinary differential equations. Solving the ordinary differential equations analytically and using a propagator matrix, the exact electro-elastic axisymmetric solution of 1D piezoelectric quasicrystal circular plate actuators under generalized elastic simply supported boundary conditions is obtained. The mechanical boundary condition is considered in the numerical examples, and subsequently the influences of the thickness-to-span ratio and stacking sequence on phonon, phason, and electric fields are presented.

## 2. Description of Actuator and Governing Equations

**Figure 1:**
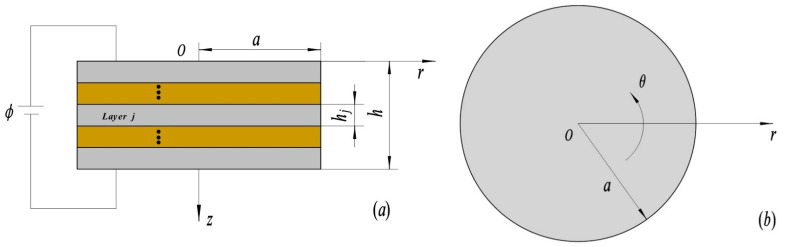
The axisymmetric 1D piezoelectric QC laminated circular plates model. (**a**) Main view; (**b**) top view.

Consider a 1D piezoelectric QC laminated circular plate model of radius *a*, *j*-th layer thickness *h_j_*, and the total thickness *h*, as shown in Figure 1. A cylindrical coordinate system (*r*, *θ*, *z*) is attached to the circular plate with the origin placed at the shaft center, the *r*, *θ*, *z*-axes are taken along the radial, circumferential, and axial of the circular plate, respectively, and the plane *z* = 0 lies on the top surface of the circular actuator. *ϕ* is electric potential, and the polarization and quasiperiodic directions of the 1D piezoelectric QCs are assumed to be along the *z*-axis.

As for the axisymmetric problem of 1D piezoelectric QC circular actuators, all the phonon–phason–electric field coupling responses are independent of *θ*. The governing equations for the axisymmetric problem of 1D hexagonal piezoelectric QC circular plate actuators in the absence of body forces and free charges can be expressed in the non-dimensionalized form as [20,25]:

(1) $$\begin{array}{l} \frac{\partial {\bar{\sigma }}_{rr}}{\partial \bar{r}}+\frac{1}{s}\frac{\partial {\bar{\sigma }}_{rz}}{\partial \bar{z}}+\frac{{\bar{\sigma }}_{rr}-{\bar{\sigma }}_{\theta \theta }}{\bar{r}}=0,\\ \frac{\partial {\bar{\sigma }}_{rz}}{\partial \bar{r}}+\frac{1}{s}\frac{\partial {\bar{\sigma }}_{zz}}{\partial \bar{z}}+\frac{{\bar{\sigma }}_{rz}}{\bar{r}}=0,\\ \frac{\partial {\bar{H}}_{zr}}{\partial \bar{r}}+\frac{1}{s}\frac{\partial {\bar{H}}_{zz}}{\partial \bar{z}}+\frac{{\bar{H}}_{zr}}{\bar{r}}=0,\\ \frac{\partial {\bar{D}}_{r}}{\partial \bar{r}}+\frac{1}{s}\frac{\partial {\bar{D}}_{z}}{\partial \bar{z}}+\frac{{\bar{D}}_{r}}{\bar{r}}=0, \end{array}$$

where ${\bar{\sigma }}_{ij}$ and ${\bar{H}}_{ij}$ (*i*, *j* = *r*, *θ*, *z*) are non-dimensionalized phonon stresses and phason stresses, respectively; ${\bar{D}}_{r}$ and ${\bar{D}}_{z}$ refer to dimensionless electric displacements; and *s* is the thickness to span ratio of the circular plate. The following dimensionless quantities are introduced in the non-dimensionalized equations:

(2) $$\begin{array}{l} \bar{r}=r/a,\;\;\;\;\;\;\bar{z}=z/h,\;\;\;\;\;\;{\bar{h}}_{j}=h_{j}/h,\;\;s=h/a,\\ \;\;{\bar{u}}_{r}=u_{r}/h,\;\;{\bar{u}}_{z}=u_{z}/h,\;\;{\bar{w}}_{z}=w_{z}/h,\\ {\bar{\sigma }}_{rr}=\sigma_{rr}/C,\;\;{\bar{\sigma }}_{\theta \theta }=\sigma_{\theta \theta }/C,\;\;{\bar{\sigma }}_{zz}=\sigma_{zz}/C,\;\\ {\bar{\sigma }}_{rz}=\sigma_{rz}/C,\;\;{\bar{H}}_{zz}=H_{zz}/C,\;\;{\bar{H}}_{zr}=H_{zr}/C,\\ {\bar{C}}_{ij}=C_{ij}/C,\;\;{\bar{R}}_{ij}=R_{ij}/C,\;\;{\bar{K}}_{ij}=K_{ij}/C,\\ \;{\bar{\xi }}_{ij}=\xi_{ij}/\xi ,\;\;{\bar{e}}_{ij}=e_{ij}/\sqrt{C\xi },\;\;{\bar{d}}_{ij}=d_{ij}/\sqrt{C\xi },\\ {\bar{D}}_{i}=D_{i}/\sqrt{C\xi },\;\;\bar{\phi }=\phi \sqrt{\xi /C}/h, \end{array}$$

in which C and ξ are elastic constants and dielectric constants, respectively. Here, C and ξ are taken as the corresponding elastic constants $C_{11}^{\left(1\right)}$ and $\xi_{33}^{\left(1\right)}$ of the first layer of the circular plate; $C_{ij}$, $R_{ij}$, and $K_{ij}$ denote phonon elastic constants, phonon–phason coupling elastic constants, and phason elastic constants, respectively; $e_{ij}$, $d_{ij}$, and $\xi_{ij}$ refer to phonon piezoelectric constants, phason piezoelectric constants, and dielectric constants, respectively; $u_{r}$ and $u_{z}$ represent phonon displacements; $w_{r}$ is phason displacements; and $D_{i}$ stands for electric displacements.

Based on the dimensionless formulations in Equation (2), the constitutive relations for 1D hexagonal piezoelectric QCs in cylindrical coordinates can be rewritten as:

(3) $$\begin{array}{l} {\bar{\sigma }}_{rr}=s{\bar{C}}_{11}\frac{\partial {\bar{u}}_{r}}{\partial \bar{r}}+s{\bar{C}}_{12}\frac{{\bar{u}}_{r}}{\bar{r}}+{\bar{C}}_{13}\frac{\partial {\bar{u}}_{z}}{\partial \bar{z}}+{\bar{R}}_{1}\frac{\partial {\bar{w}}_{z}}{\partial \bar{z}}+{\bar{e}}_{31}\frac{\partial \bar{\phi }}{\partial \bar{z}},\\ {\bar{\sigma }}_{\theta \theta }=s{\bar{C}}_{12}\frac{\partial {\bar{u}}_{r}}{\partial \bar{r}}+s{\bar{C}}_{11}\frac{{\bar{u}}_{r}}{\bar{r}}+{\bar{C}}_{13}\frac{\partial {\bar{u}}_{z}}{\partial \bar{z}}+{\bar{R}}_{1}\frac{\partial {\bar{w}}_{z}}{\partial \bar{z}}+{\bar{e}}_{31}\frac{\partial \bar{\phi }}{\partial \bar{z}},\\ {\bar{\sigma }}_{zz}=s{\bar{C}}_{13}\frac{\partial {\bar{u}}_{r}}{\partial \bar{r}}+s{\bar{C}}_{13}\frac{{\bar{u}}_{r}}{\bar{r}}+{\bar{C}}_{33}\frac{\partial {\bar{u}}_{z}}{\partial \bar{z}}+{\bar{R}}_{2}\frac{\partial {\bar{w}}_{z}}{\partial \bar{z}}+{\bar{e}}_{33}\frac{\partial \bar{\phi }}{\partial \bar{z}},\\ {\bar{\sigma }}_{rz}={\bar{\sigma }}_{zr}={\bar{C}}_{44}\left(s\frac{\partial {\bar{u}}_{z}}{\partial \bar{r}}+\frac{\partial {\bar{u}}_{r}}{\partial \bar{z}}\right)+s{\bar{R}}_{3}\frac{\partial {\bar{w}}_{z}}{\partial \bar{r}}+s{\bar{e}}_{15}\frac{\partial \bar{\phi }}{\partial \bar{r}},\\ {\bar{H}}_{zr}={\bar{R}}_{3}\left(s\frac{\partial {\bar{u}}_{z}}{\partial \bar{r}}+\frac{\partial {\bar{u}}_{r}}{\partial \bar{z}}\right)+s{\bar{K}}_{2}\frac{\partial {\bar{w}}_{z}}{\partial \bar{r}}+s{\bar{d}}_{15}\frac{\partial \bar{\phi }}{\partial \bar{r}},\\ {\bar{H}}_{zz}=s{\bar{R}}_{1}\frac{\partial {\bar{u}}_{r}}{\partial \bar{r}}+s{\bar{R}}_{1}\frac{{\bar{u}}_{r}}{\bar{r}}+{\bar{R}}_{2}\frac{\partial {\bar{u}}_{z}}{\partial \bar{z}}+{\bar{K}}_{1}\frac{\partial {\bar{w}}_{z}}{\partial \bar{z}}+{\bar{d}}_{33}\frac{\partial \bar{\phi }}{\partial \bar{z}},\\ {\bar{D}}_{r}={\bar{e}}_{15}\left(s\frac{\partial {\bar{u}}_{z}}{\partial \bar{r}}+\frac{\partial {\bar{u}}_{r}}{\partial \bar{z}}\right)+s{\bar{d}}_{15}\frac{\partial {\bar{w}}_{z}}{\partial \bar{r}}-s{\bar{\xi }}_{11}\frac{\partial \bar{\phi }}{\partial \bar{r}},\\ {\bar{D}}_{z}=s{\bar{e}}_{31}\frac{\partial {\bar{u}}_{r}}{\partial \bar{r}}+s{\bar{e}}_{31}\frac{{\bar{u}}_{r}}{\bar{r}}+{\bar{e}}_{33}\frac{\partial {\bar{u}}_{z}}{\partial \bar{z}}+{\bar{d}}_{33}\frac{\partial {\bar{w}}_{z}}{\partial \bar{z}}-{\bar{\xi }}_{33}\frac{\partial \bar{\phi }}{\partial \bar{z}}. \end{array}$$

## 3. State Formulation and Hankel Transform

For layer *j* of the 1D piezoelectric QC laminated circular actuators, shown in Figure 1, if ${\bar{u}}_{r}$, ${\bar{\sigma }}_{zz}$, ${\bar{H}}_{zz}$, ${\bar{D}}_{z}$, ${\bar{\sigma }}_{rz}$, ${\bar{u}}_{z}$, ${\bar{w}}_{z}$, and $\bar{\phi }$ are set as state variables, the state space equation for layer *j* can be obtained from Equations (2) and (3) as:

(4) $$\frac{\partial {\bar{R}}_{j}\left(\bar{r},\;\;\;\;\bar{z}\right)}{\partial \bar{z}}=\left[\begin{array}{cc} 0 & A_{j}\\ B_{j} & 0 \end{array}\right]{\bar{R}}_{j}\left(\bar{r},\;\;\;\;\bar{z}\right),$$

where

(5) $${\bar{R}}_{j}={\left[\begin{array}{ccccccc} {\bar{u}}_{r} & {\bar{\sigma }}_{zz} & {\bar{H}}_{zz} & {\bar{D}}_{z} & {\bar{\sigma }}_{rz} & {\bar{u}}_{z} & \begin{array}{cc} {\bar{w}}_{z} & \bar{\phi } \end{array} \end{array}\right]}^{T},$$

and the superscript “*T*” represents the matrix transpose. The matrices $A_{j}$ and $B_{j}$ take the form:

(6) $$\begin{array}{l} A_{j}=\left[\begin{array}{cc} A_{j1} & A_{j2}\\ A_{j3} & A_{j4} \end{array}\right],\;\;\;\;\;\;A_{j1}=\left[\begin{array}{cc} \alpha_{1} & -s\frac{\partial }{\partial \bar{r}}\\ -s\left(\frac{1}{\bar{r}}+\frac{\partial }{\partial \bar{r}}\right) & 0 \end{array}\right],\;\;\;\;\;\;A_{j2}=\left[\begin{array}{cc} -\alpha_{2}\frac{\partial }{\partial \bar{r}} & -\alpha_{3}\frac{\partial }{\partial \bar{r}}\\ 0 & 0 \end{array}\right],\\ A_{j3}=\left[\begin{array}{cc} -\alpha_{2}\left(\frac{1}{\bar{r}}+\frac{\partial }{\partial \bar{r}}\right) & 0\\ -\alpha_{3}\left(\frac{1}{\bar{r}}+\frac{\partial }{\partial \bar{r}}\right) & 0 \end{array}\right],\;\;\;\;\;\;A_{j4}=\left[\begin{array}{cc} \alpha_{4}\left(\frac{1}{\bar{r}}\frac{\partial }{\partial \bar{r}}+\frac{\partial^{2}}{\partial {\bar{r}}^{2}}\right) & \alpha_{5}\left(\frac{1}{\bar{r}}\frac{\partial }{\partial \bar{r}}+\frac{\partial^{2}}{\partial {\bar{r}}^{2}}\right)\\ \alpha_{5}\left(\frac{1}{\bar{r}}\frac{\partial }{\partial \bar{r}}+\frac{\partial^{2}}{\partial {\bar{r}}^{2}}\right) & \alpha_{6}\left(\frac{1}{\bar{r}}\frac{\partial }{\partial \bar{r}}+\frac{\partial^{2}}{\partial {\bar{r}}^{2}}\right) \end{array}\right], \end{array}$$

and

(7) $$\begin{array}{l} B_{j}=\left[\begin{array}{cc} B_{j1} & B_{j2}\\ B_{j3} & B_{j4} \end{array}\right],\;\;\;\;\;\;B_{j1}=\left[\begin{array}{cc} \beta_{1}\left(\frac{\partial^{2}}{\partial {\bar{r}}^{2}}+\frac{1}{\bar{r}}\frac{\partial }{\partial \bar{r}}-\frac{1}{{\bar{r}}^{2}}\right) & \beta_{2}\frac{\partial }{\partial \bar{r}}\\ \beta_{2}\left(\frac{1}{\bar{r}}+\frac{\partial }{\partial \bar{r}}\right) & \beta_{5} \end{array}\right],\;\;\;\;\;\;B_{j2}=\left[\begin{array}{cc} \beta_{3}\frac{\partial }{\partial \bar{r}} & \beta_{4}\frac{\partial }{\partial \bar{r}}\\ \beta_{6} & \beta_{7} \end{array}\right],\\ B_{j3}=\left[\begin{array}{cc} \beta_{3}\left(\frac{1}{\bar{r}}+\frac{\partial }{\partial \bar{r}}\right) & \beta_{6}\\ \beta_{4}\left(\frac{1}{\bar{r}}+\frac{\partial }{\partial \bar{r}}\right) & \beta_{7} \end{array}\right],\;\;\;\;\;\;B_{j4}=\left[\begin{array}{cc} \beta_{8} & \beta_{9}\\ \beta_{9} & \beta_{10} \end{array}\right]. \end{array}$$

We also obtain other derived variables as

(8) $$\begin{array}{l} {\bar{\sigma }}_{rr}\left(\bar{r},\;\;\;\;\bar{z}\right)=-\frac{1}{s}\left[\beta_{11}\frac{{\bar{u}}_{r}\left(\bar{r},\;\;\;\;\bar{z}\right)}{\bar{r}}+\beta_{1}\frac{\partial {\bar{u}}_{r}\left(\bar{r},\;\;\;\;\bar{z}\right)}{\partial \bar{r}}+\beta_{2}{\bar{\sigma }}_{zz}\left(\bar{r},\;\;\;\;\bar{z}\right)+\;\beta_{3}{\bar{H}}_{zz}\left(\bar{r},\;\;\;\;\bar{z}\right)+\beta_{4}{\bar{D}}_{z}\left(\bar{r},\;\;\;\;\bar{z}\right)\right],\\ {\bar{\sigma }}_{\theta \theta }\left(\bar{r},\;\;\;\;\bar{z}\right)=-\frac{1}{s}\left[\beta_{1}\frac{{\bar{u}}_{r}\left(\bar{r},\;\;\;\;\bar{z}\right)}{\bar{r}}+\beta_{11}\frac{\partial {\bar{u}}_{r}\left(\bar{r},\;\;\;\;\bar{z}\right)}{\partial \bar{r}}+\beta_{2}{\bar{\sigma }}_{zz}\left(\bar{r},\;\;\;\;\bar{z}\right)+\;\beta_{3}{\bar{H}}_{zz}\left(\bar{r},\;\;\;\;\bar{z}\right)+\beta_{4}{\bar{D}}_{z}\left(\bar{r},\;\;\;\;\bar{z}\right)\right],\\ {\bar{D}}_{r}\left(\bar{r},\;\;\;\;\bar{z}\right)=\frac{1}{s}\left[\alpha_{3}{\bar{\sigma }}_{rz}\left(\bar{r},\;\;\;\;\bar{z}\right)-\alpha_{5}\frac{\partial {\bar{w}}_{z}\left(\bar{r},\;\;\;\;\bar{z}\right)}{\partial \bar{r}}-\alpha_{6}\frac{\partial \bar{\phi }\left(\bar{r},\;\;\;\;\bar{z}\right)}{\partial \bar{r}}\right],\\ {\bar{H}}_{zr}\left(\bar{r},\;\;\;\;\bar{z}\right)=\;\frac{1}{s}\left[\alpha_{2}{\bar{\sigma }}_{rz}\left(\bar{r},\;\;\;\;\bar{z}\right)-\alpha_{4}\frac{\partial {\bar{w}}_{z}\left(\bar{r},\;\;\;\;\bar{z}\right)}{\partial \bar{r}}-\alpha_{5}\frac{\partial \bar{\phi }\left(\bar{r},\;\;\;\;\bar{z}\right)}{\partial \bar{r}}\right], \end{array}$$

in which the definition of parameters $\alpha_{m}$ (*m* = 1, 2, …, 6) and $\beta_{n}$ (*n* = 1, 2, …, 11) are presented in Appendix A. 

To deal with the axisymmetric problem of 1D piezoelectric QC circular plates, the finite Hankel transform is introduced, which is defined as:

(9) $$J_{\mu }\left[f\left(\bar{r},\;\;\;\;\bar{z}\right)\right]=\int_{0}^{1}\bar{r}f\left(\bar{r},\;\;\;\;\bar{z}\right)J_{\mu }\left(k\bar{r}\right)d\bar{r},$$

where $J_{\mu }\left(k\bar{r}\right)$ is the *μ*-th order Bessel function of the first kind. According to the definition in Equation (9), the state space vector can be represented as:

(10) $$R_{j}\left(k,\;\;\;\;\bar{z}\right)={\left[\begin{array}{c} U_{r}\left(k,\;\;\;\;\bar{z}\right)\\ S\left(k,\;\;\;\;\bar{z}\right)\\ H\left(k,\;\;\;\;\bar{z}\right)\\ D\left(k,\;\;\;\;\bar{z}\right)\\ T\left(k,\;\;\;\;\bar{z}\right)\\ U_{z}\left(k,\;\;\;\;\bar{z}\right)\\ W\left(k,\;\;\;\;\bar{z}\right)\\ F\left(k,\;\;\;\;\bar{z}\right) \end{array}\right]}_{j}={\left[\begin{array}{c} J_{1}\left[{\bar{u}}_{r}\left(\bar{r},\;\;\;\;\bar{z}\right)\right]\\ J_{0}\left[{\bar{\sigma }}_{zz}\left(\bar{r},\;\;\;\;\bar{z}\right)\right]\\ J_{0}\left[{\bar{H}}_{zz}\left(\bar{r},\;\;\;\;\bar{z}\right)\right]\\ J_{0}\left[{\bar{D}}_{z}\left(\bar{r},\;\;\;\;\bar{z}\right)\right]\\ J_{1}\left[{\bar{\sigma }}_{rz}\left(\bar{r},\;\;\;\;\bar{z}\right)\right]\\ J_{0}\left[{\bar{u}}_{z}\left(\bar{r},\;\;\;\;\bar{z}\right)\right]\\ J_{0}\left[{\bar{w}}_{z}\left(\bar{r},\;\;\;\;\bar{z}\right)\right]\\ J_{0}\left[\bar{\phi }\left(\bar{r},\;\;\;\;\bar{z}\right)\right] \end{array}\right]}_{j}.$$

By using Equations (9) and (10), the finite Hankel transformation is performed on the state equation shown in Equation (4) leading to:

(11) $$\frac{\partial R_{j}\left(k,\;\;\;\;\bar{z}\right)}{\partial \bar{z}}=K_{j}\left(k\right)R_{j}\left(k,\;\;\;\;\bar{z}\right)+Q_{j}\left(k,\;\;\;\;\bar{z}\right),$$

in which the matrix $K_{j}$ is

(12) $$\begin{array}{c} K_{j}\left(k\right)=\left[\begin{array}{cc} 0 & K_{j1}\left(k\right)\\ K_{j2}\left(k\right) & 0 \end{array}\right],\\ K_{j1}\left(k\right)=\left[\begin{array}{cccc} \alpha_{1} & sk & \alpha_{2}k & \alpha_{3}k\\ -sk & 0 & 0 & 0\\ -\alpha_{2}k & 0 & -\alpha_{4}k^{2} & -\alpha_{5}k^{2}\\ -\alpha_{3}k & 0 & -\alpha_{5}k^{2} & -\alpha_{6}k^{2} \end{array}\right],\;\;\;\;\;\;K_{j2}\left(k\right)=\left[\begin{array}{cccc} -\beta_{1}k^{2} & -\beta_{2}k & -\beta_{3}k & -\beta_{4}k\\ \beta_{2}k & \beta_{5} & \beta_{6} & \beta_{7}\\ \beta_{3}k & \beta_{6} & \beta_{8} & \beta_{9}\\ \beta_{4}k & \beta_{7} & \beta_{9} & \beta_{10} \end{array}\right], \end{array}$$

and the matrix $Q_{j}$ is

(13) $$\begin{array}{l} Q_{j}\left(k,\;\;\;\;\bar{z}\right)={\left[Q_{j1}\;\;\;\;\;\;Q_{j2}\;\;\;\;\;Q_{j3}\;\;\;\;\;Q_{j4}\;\;\;\;\;Q_{j5}\;\;\;\;\;Q_{j6}\;\;\;\;\;Q_{j7}\;\;\;\;\;Q_{j8}\right]}^{T},\\ Q_{j1}=-s{\bar{u}}_{z}\left(1,\bar{z}\right)J_{1}\left(k\right)-\alpha_{2}{\bar{w}}_{z}\left(1,\bar{z}\right)J_{1}\left(k\right)-\alpha_{3}\bar{\phi }\left(1,\bar{z}\right)J_{1}\left(k\right),\\ Q_{j2}=-s{\bar{\sigma }}_{rz}\left(1,\bar{z}\right)J_{0}(k),\\ Q_{j3}=\left[-\alpha_{2}{\bar{\sigma }}_{rz}\left(1,\bar{z}\right)+\alpha_{4}\frac{\partial {\bar{w}}_{z}\left(1,\bar{z}\right)}{\partial \bar{r}}+\alpha_{5}\frac{\partial \bar{\phi }\left(1,\bar{z}\right)}{\partial \bar{r}}\right]J_{0}\left(k\right)\;\;+\left[\alpha_{4}k{\bar{w}}_{z}\left(1,\bar{z}\right)+\alpha_{5}k\bar{\phi }\left(1,\bar{z}\right)\right]J_{1}\left(k\right),\\ Q_{j4}=\left[-\alpha_{3}{\bar{\sigma }}_{rz}\left(1,\bar{z}\right)+\alpha_{5}\frac{\partial {\bar{w}}_{z}\left(1,\bar{z}\right)}{\partial \bar{r}}+\alpha_{6}\frac{\partial \bar{\phi }\left(1,\bar{z}\right)}{\partial \bar{r}}\right]J_{0}\left(k\right)\;+\left[\alpha_{5}k{\bar{w}}_{z}\left(1,\bar{z}\right)+\alpha_{6}k\bar{\phi }\left(1,\bar{z}\right)\right]J_{1}\left(k\right),\\ Q_{j5}=[\beta_{1}\frac{\partial {\bar{u}}_{r}\left(1,\bar{z}\right)}{\partial \bar{r}}+\beta_{1}{\bar{u}}_{r}\left(1,\bar{z}\right)+\beta_{2}{\bar{\sigma }}_{zz}\left(1,\bar{z}\right)+\beta_{3}{\bar{H}}_{zz}\left(1,\bar{z}\right)+\beta_{4}{\bar{D}}_{z}\left(1,\bar{z}\right)]J_{1}\left(k\right)-\beta_{1}k{\bar{u}}_{r}\left(1,\bar{z}\right)J_{0}\left(k\right),\\ Q_{j6}=\beta_{2}{\bar{u}}_{r}\left(1,\bar{z}\right)J_{0}\left(k\right),\\ Q_{j7}=\beta_{3}{\bar{u}}_{r}\left(1,\bar{z}\right)J_{0}\left(k\right),\\ Q_{j8}=\beta_{4}{\bar{u}}_{r}\left(1,\bar{z}\right)J_{0}\left(k\right). \end{array}$$

The following formulations can be obtained by letting $\bar{r}=1$ in Equation (8):

(14) $$\begin{array}{l} s{\bar{D}}_{r}\left(1,\bar{z}\right)=\alpha_{3}{\bar{\sigma }}_{rz}\left(1,\bar{z}\right)-\alpha_{5}\frac{\partial {\bar{w}}_{z}\left(1,\bar{z}\right)}{\partial \bar{r}}-\alpha_{6}\frac{\partial \bar{\phi }\left(1,\bar{z}\right)}{\partial \bar{r}},\\ s{\bar{\sigma }}_{rr}\left(1,\bar{z}\right)=-\beta_{11}{\bar{u}}_{r}\left(1,\bar{z}\right)-\beta_{1}\frac{\partial {\bar{u}}_{r}\left(1,\bar{z}\right)}{\partial \bar{r}}\;-\beta_{2}{\bar{\sigma }}_{zz}\left(1,\bar{z}\right)-\beta_{3}{\bar{H}}_{zz}\left(1,\bar{z}\right)-\beta_{4}{\bar{D}}_{z}\left(1,\bar{z}\right),\\ s{\bar{H}}_{zr}\left(1,\bar{z}\right)=\alpha_{2}{\bar{\sigma }}_{rz}\left(1,\bar{z}\right)-\alpha_{4}\frac{\partial {\bar{w}}_{z}\left(1,\bar{z}\right)}{\partial \bar{r}}-\alpha_{5}\frac{\partial \bar{\phi }\left(1,\bar{z}\right)}{\partial \bar{r}}. \end{array}$$

By substituting Equation (14) into Equation (13), some submatrices in the matrix $Q_{j}$ can be simplified as:

(15) $$\begin{array}{l} Q_{j3}=-s{\bar{H}}_{zr}\left(1,\bar{z}\right)J_{0}\left(k\right)+\alpha_{4}k{\bar{w}}_{z}\left(1,\bar{z}\right)J_{1}\left(k\right)\;+\alpha_{5}k\bar{\phi }\left(1,\bar{z}\right)J_{1}\left(k\right),\\ Q_{j4}=-s{\bar{D}}_{r}\left(1,\bar{z}\right)J_{0}\left(k\right)+\alpha_{5}k{\bar{w}}_{z}\left(1,\bar{z}\right)J_{1}\left(k\right)+\alpha_{6}k\bar{\phi }\left(1,\bar{z}\right)J_{1}\left(k\right),\\ Q_{j5}=({\bar{C}}_{12}-{\bar{C}}_{11})s^{2}{\bar{u}}_{r}\left(1,\bar{z}\right)J_{1}\left(k\right)-s{\bar{\sigma }}_{rr}\left(1,\bar{z}\right)J_{1}\left(k\right)\;-\beta_{1}k{\bar{u}}_{r}\left(1,\bar{z}\right)J_{0}\left(k\right). \end{array}$$

By extending the generalized elastic simple support boundary condition of the circular plate proposed by Ding et al. [19] to 1D piezoelectric QC circular plates, we obtain:

(16) $${\bar{u}}_{z}\left(1,\bar{z}\right)=0,\;\;\;\;\;\;{\bar{w}}_{z}\left(1,\bar{z}\right)=0,\;\;\;\;\;\;\bar{\phi }\left(1,\bar{z}\right)=0\;,\;\;\;\;\;\;\left[\left({\bar{C}}_{11}-{\bar{C}}_{12}\right)s{\bar{u}}_{r}\left(1,\bar{z}\right)+{\bar{\sigma }}_{rr}\left(1,\bar{z}\right)\right]=0\;\;\mathrm{and}\;\;J_{0}\left(k\right)=0.$$

Under the above boundary condition, the matrix $Q_{j}\left(k,\;\;\;\;\bar{z}\right)$ in Equation (11) is eliminated, and thus Equation (11) becomes a homogeneous equation:

(17) $$\frac{\partial R_{j}\left(k,\;\;\;\;\bar{z}\right)}{\partial \bar{z}}=K_{j}\left(k\right)R_{j}\left(k,\;\;\;\;\bar{z}\right).$$

The solution of the ordinary differential equation shown in Equation (17) can be written as:

(18) $$R_{j}\left(k,\;\;\;\;\bar{z}\right)=T_{j}\left(k,\;\;\;\;\bar{z}\right)R_{j}\left(k,\;\;\;\;0\right),$$

where the propagator matrix $T_{j}$ is

(19) $$T_{j}\left(k,\;\;\;\;\bar{z}\right)=\exp\left[K_{j}\left(k\right)\bar{z}\right].$$

It is assumed that there is a perfect connection interface between two adjacent layers of circular laminates. Taking *z* = *z_j_* corresponding to layer *j* and layer *j* + 1 as an example, the state variables meet the following relationship:

(20) $$R_{j+1}(k,\;\;\;\;0)=R_{j}(k,\;\;\;\;{\bar{h}}_{j}).$$

Combining with the interface connection conditions and propagator matrix, the state variables of the circular plate at any *z*-level can be expressed as:

(21) $$R_{p}(k,\;\;\;\;{\bar{z}}_{p})=P(k)R_{1}(k,\;\;\;\;0),$$

where

(22) $$P(k)=\prod_{j=1}^{N}T_{j}(k,\;\;\;\;{\bar{h}}_{j}).$$

## 4. Boundary Condition and Its Solutions

Considering that the top and bottom surfaces of the circular actuator are subjected to mechanical loading, and the dimensionless mechanical boundary conditions can be expressed as

(23) $${\bar{\sigma }}_{zz}(\bar{r},0)=\sigma_{0}(\bar{r}),\;\;\;\;\;\;\;\;{\bar{\sigma }}_{zz}(\bar{r},1)=\sigma_{1}(\bar{r}),$$

and the dimensionless electrical boundary condition is:

(24) $${\bar{D}}_{z}(\bar{r},0)={\bar{D}}_{z}(\bar{r},1)=0.$$

According to the definition of finite Hankel transformations in Equation (9), the loading conditions in Equation (23) can be written as:

(25) $$\begin{array}{l} S(k,0)=\int_{0}^{1}\bar{r}\sigma_{0}(\bar{r})J_{0}(k\bar{r})d\bar{r},\\ S(k,1)=\int_{0}^{1}\bar{r}\sigma_{1}(\bar{r})J_{0}(k\bar{r})d\bar{r}. \end{array}$$

By virtue of Equations (21) and (23), the state space vector $R_{1}\left(k,\;\;\;\;0\right)$ on the top surface is obtained:

(26) $$\left[\begin{array}{c} U_{r}(k,0)\\ U_{z}(k,0)\\ W(k,0)\\ F(k,0) \end{array}\right]={\left[\begin{array}{cccc} P_{21} & P_{26} & P_{27} & P_{28}\\ P_{31} & P_{36} & P_{37} & P_{38}\\ P_{41} & P_{46} & P_{47} & P_{48}\\ P_{51} & P_{56} & P_{57} & P_{58} \end{array}\right]}^{-1}\left[\begin{array}{c} S(k,1)\\ H(k,1)\\ D(k,1)\\ T(k,1) \end{array}\right]-{\left[\begin{array}{cccc} P_{21} & P_{26} & P_{27} & P_{28}\\ P_{31} & P_{36} & P_{37} & P_{38}\\ P_{41} & P_{46} & P_{47} & P_{48}\\ P_{51} & P_{56} & P_{57} & P_{58} \end{array}\right]}^{-1}\left[\begin{array}{cccc} P_{22} & P_{23} & P_{24} & P_{25}\\ P_{32} & P_{33} & P_{34} & P_{35}\\ P_{42} & P_{43} & P_{44} & P_{45}\\ P_{52} & P_{53} & P_{54} & P_{55} \end{array}\right]\times \left[\begin{array}{c} S(k,0)\\ H(k,0)\\ D(k,0)\\ T(k,0) \end{array}\right].$$

With the aid of Equations (18) and (20), we can obtain the state space vector $R_{j}\left(k,\;\;\;\;\bar{z}\right)$ at any *j*-th layer. Based on the inverse Hankel transform [26], the dimensionless physical quantities of phonon, phason, and electric fields are obtained:

(27) $$\begin{array}{l} {\bar{u}}_{r}(\bar{r},\bar{z})=2\sum_{i}U_{r}(k_{i},\bar{z})\frac{J_{1}(k_{i}\bar{r})}{{\left[J_{1}(k_{i})\right]}^{2}},\;\;\;\;\;\;{\bar{\sigma }}_{zz}(\bar{r},\bar{z})=2\sum_{i}S(k_{i},\bar{z})\frac{J_{0}(k_{i}\bar{r})}{{\left[J_{1}(k_{i})\right]}^{2}},\\ {\bar{H}}_{zz}(\bar{r},\bar{z})=2\sum_{i}H(k_{i},\bar{z})\frac{J_{0}(k_{i}\bar{r})}{{\left[J_{1}(k_{i})\right]}^{2}},\;\;\;\;\;\;{\bar{D}}_{z}(\bar{r},\bar{z})=2\sum_{i}D(k_{i},\bar{z})\frac{J_{0}(k_{i}\bar{r})}{{\left[J_{1}(k_{i})\right]}^{2}},\\ {\bar{\sigma }}_{rz}(\bar{r},\bar{z})=2\sum_{i}T(k_{i},\bar{z})\frac{J_{1}(k_{i}\bar{r})}{{\left[J_{1}(k_{i})\right]}^{2}},\;\;\;\;\;\;{\bar{u}}_{z}(\bar{r},\bar{z})=2\sum_{i}U_{z}(k_{i},\bar{z})\frac{J_{0}(k_{i}\bar{r})}{{\left[J_{1}(k_{i})\right]}^{2}},\\ {\bar{w}}_{z}(\bar{r},\bar{z})=2\sum_{i}W(k_{i},\bar{z})\frac{J_{0}(k_{i}\bar{r})}{{\left[J_{1}(k_{i})\right]}^{2}},\;\;\;\;\;\;\bar{\phi }(\bar{r},\bar{z})=2\sum_{i}F(k_{i},\bar{z})\frac{J_{0}(k_{i}\bar{r})}{{\left[J_{1}(k_{i})\right]}^{2}}, \end{array}$$

and

(28) $$\begin{array}{l} {\bar{\sigma }}_{rr}(\bar{r},\bar{z})=(C_{12}-C_{11})s\frac{{\bar{u}}_{r}(\bar{r},\bar{z})}{\bar{r}}-\frac{2\beta_{1}}{s}\sum_{i}k_{i}U_{r}(k_{i},\bar{z})\frac{J_{0}(k_{i}\bar{r})}{{\left[J_{1}(k_{i})\right]}^{2}}-\frac{\beta_{2}}{s}{\bar{\sigma }}_{zz}(\bar{r},\bar{z})\;-\frac{\beta_{3}}{s}\;{\bar{H}}_{zz}(\bar{r},\bar{z})-\frac{\beta_{4}}{s}{\bar{D}}_{z}(\bar{r},\bar{z}),\\ {\bar{\sigma }}_{\theta \theta }(\bar{r},\bar{z})=-(C_{12}-C_{11})s\frac{{\bar{u}}_{r}(\bar{r},\bar{z})}{\bar{r}}\;\;-\frac{2\beta_{11}}{s}\sum_{i}k_{i}U_{r}(k_{i},\bar{z})\frac{J_{0}(k_{i}\bar{r})}{{\left[J_{1}(k_{i})\right]}^{2}}-\frac{\beta_{2}}{s}{\bar{\sigma }}_{zz}(\bar{r},\bar{z})-\frac{\beta_{3}}{s}\;{\bar{H}}_{zz}(\bar{r},\bar{z})-\frac{\beta_{4}}{s}{\bar{D}}_{z}(\bar{r},\bar{z}),\\ {\bar{D}}_{r}(\bar{r},\bar{z})=\frac{\alpha_{3}}{s}{\bar{\sigma }}_{rz}(\bar{r},\bar{z})+\frac{2\alpha_{5}}{s}\sum_{i}k_{i}W(k_{i},\bar{z})\frac{J_{1}(k_{i}\bar{r})}{{\left[J_{1}(k_{i})\right]}^{2}}+\frac{2\alpha_{6}}{s}\sum_{i}k_{i}F(k_{i},\bar{z})\frac{J_{1}(k_{i}\bar{r})}{{\left[J_{1}(k_{i})\right]}^{2}},\\ {\bar{H}}_{zr}(\bar{r},\bar{z})=\frac{\alpha_{2}}{s}{\bar{\sigma }}_{rz}(\bar{r},\bar{z})+\frac{2\alpha_{4}}{s}\sum_{i}k_{i}W(k_{i},\bar{z})\frac{J_{1}(k_{i}\bar{r})}{{\left[J_{1}(k_{i})\right]}^{2}}+\frac{2\alpha_{5}}{s}\sum_{i}k_{i}F(k_{i},\bar{z})\frac{J_{1}(k_{i}\bar{r})}{{\left[J_{1}(k_{i})\right]}^{2}}. \end{array}$$

## 5. Numerical Examples

Consider a 1D piezoelectric QC circular actuator subjected to mechanical loading, which is under the generalized elastic simply supported boundary conditions. Three-layered circular plates with different stacking sequences are considered in this paper. They are piezoelectric materials/piezoelectric QCs/piezoelectric materials (P/Q/P), piezoelectric QCs/piezoelectric materials/piezoelectric QCs (Q/P/Q), and piezoelectric materials/piezoelectric materials/piezoelectric materials (P/P/P), respectively. The corresponding material parameters of 1D hexagonal piezoelectric QCs [12] and piezoelectric materials (PZT4) [19] are tabulated in Table 1.

### 5.1. Verification of the Present Method

**Figure 2:**
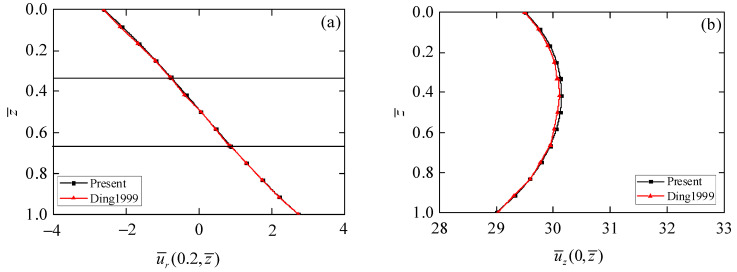
Comparison results of phonon displacements. (**a**) Radial displacement ${\bar{u}}_{r}$; (**b**) axial displacement ${\bar{u}}_{z}$.

In order to verify the correctness of the exact solution obtained in this paper, we considered a piezoelectric circular plate with thickness to span ratio *s* = 0.4, whose top surface is subjected to the mechanical loading ${\bar{\sigma }}_{zz}(\bar{r},0)=\sigma_{0}(\bar{r})=-1$, which was investigated by Ding et al. [27]. The exact solution presented in this paper is reduced to the piezoelectric materials. Comparison results of radial displacement ${\bar{u}}_{r}\left(0.2,\;\;\;\bar{z}\right)$ and axial displacement ${\bar{u}}_{z}\left(0,\;\;\;\bar{z}\right)$ are shown in Figure 2. It can be observed from Figure 2 that the radial and axial displacements obtained in this paper agree well with those in [27], which can prove the correctness of exact axisymmetric solutions for 1D piezoelectric circular plates.

### 5.2. Effect of the Thickness to Span Ratio on the Circular Actuator

Consider a piezoelectric circular plate with elastic simply supported boundary conditions, whose top surface is subjected to mechanical loading. Let the boundary condition expressions be ${\bar{\sigma }}_{zz}(\bar{r},0)=\sigma_{0}(\bar{r})=-J_{0}\left(k_{1}\bar{r}\right)$ and ${\bar{\sigma }}_{zz}(\bar{r},1)=\sigma_{1}(\bar{r})=0$, where *k*_1_ = 2.404826 is the first zero point of $J_{0}\left(k\right)$ under the generalized elastic simply supported boundary conditions. We present the influence of thickness to span ratio *s* on the axial phonon displacements ${\bar{u}}_{z}\left(0,\;\;\;0.5\right)$ of sandwich circular plates with different stacking sequences.

It can be observed from Table 2 that that the *z*-direction phonon displacements decrease with an increasing thickness to span ratio. Furthermore, for any given thickness to span ratio *s*, the value of ${\bar{u}}_{z}$ for the Q/P/Q circular plate is larger than those in the other two laminated circular plates. This phenomenon indicates that the laminated actuator with outer layers of QCs has the largest deformation, which reflects the better electro-mechanical coupling effect. In addition, the difference between the values of ${\bar{u}}_{z}$ for different stacking sequences is small when *s* is relatively large, but the difference becomes larger with the decrease of *s*. The phenomenon concluded can provide a theoretical reference for engineers designing piezoelectric actuators made of QCs.

### 5.3. Effect of the Stacking Sequence on the Circular Actuator

In this section, numerical examples are performed to reveal the influence of stacking sequence on the axisymmetric bending behaviors of the present circular plate model. Consider a 1D piezoelectric QC circular actuator with the generalized elastic simply supported boundary condition, whose top surface is subjected to mechanical loading, as mentioned in Section 5.2, and the thickness to span ratio *s* is chosen as 0.5.

**Figure 3:**
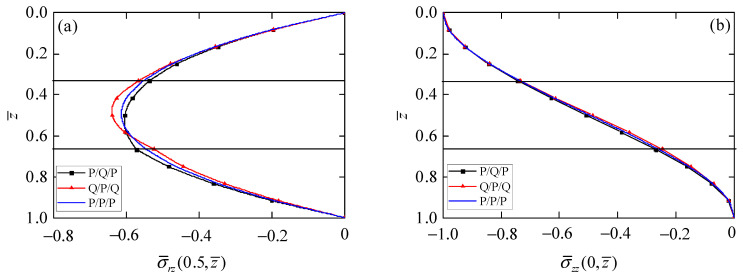

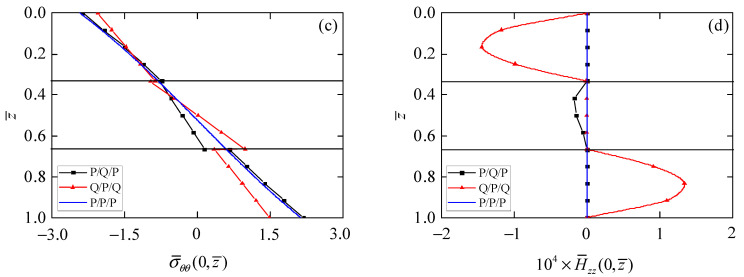
Influence of stacking sequence on phonon and phason stresses. (**a**) Phonon stress ${\bar{\sigma }}_{rz}$; (**b**) phonon stress ${\bar{\sigma }}_{zz}$; (**c**) phonon stress ${\bar{\sigma }}_{\theta \theta }$; (**d**) phason stress ${\bar{H}}_{zz}$.

**Figure 5:**
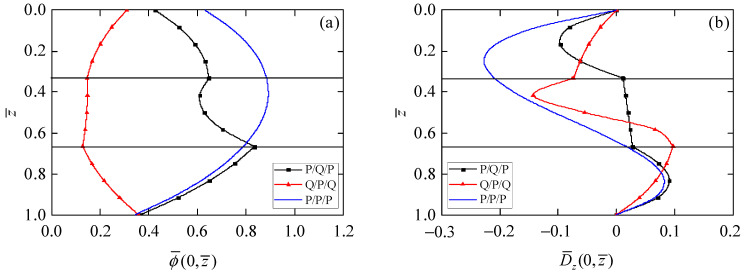
Influence of stacking sequence on electric field. (**a**) Electric potential $\bar{\phi }$; (**b**) electric displacement ${\bar{D}}_{z}$.

By observing the values of phonon stress ${\bar{\sigma }}_{rz}$ in Figure 3a, phonon stress ${\bar{\sigma }}_{zz}$ in Figure 3b, phason stress ${\bar{H}}_{zz}$ in Figure 3d, and electric displacement ${\bar{D}}_{z}$ in Figure 5b on the top and bottom surfaces of the circular plate, all meet the applied boundary conditions. It can be again observed from those results that the presented exact axisymmetric solution of 1D piezoelectric circular plates is correct.

Figure 3 presents the influence of the stacking sequence of laminated circular plates on the stress components in phonon and phason fields. Phonon stress ${\bar{\sigma }}_{rz}$ (Figure 3a) distributes as a parabolic function in the laminated circular plates with different stacking sequences. The point of similarity is that the maximum values of ${\bar{\sigma }}_{rz}$ for all laminated circular plates are at $\bar{z}$ = 0.5, because the material parameters are symmetrical along the middle plane of the laminated circular plates. The point of difference is that the maximum value of ${\bar{\sigma }}_{rz}$ is largest in the Q/P/Q circular plate and smallest in the P/Q/P circular plate. There is little difference for phonon stress ${\bar{\sigma }}_{zz}$ (Figure 3b) in laminated circular plates with different stacking sequences since the same loading is applied on the top surface of the laminated circular plates. As can be seen from Figure 3c, phonon stress ${\bar{\sigma }}_{\theta \theta }$ in the P/P/P circular plate is continuous at the interface, but it is discontinuous at the interface of the Q/P/Q and P/Q/P circular plates, mainly due to the change of material parameters for each layer. In addition, compared with the value of ${\bar{\sigma }}_{\theta \theta }$ on the top and bottom surfaces in the P/Q/P circular plate, their values are smaller when the QC layer is chosen as the outer layer of the laminated circular plates, which may reduce the risk of delamination of the laminated actuators. Phason stress ${\bar{H}}_{zz}$ is zero in the piezoelectric layer, but follows the parabolic function distribution in the QC layer. The value of ${\bar{H}}_{zz}$ in the Q/P/Q circular plate is larger than that in the P/Q/P circular plate, because there are more QCs in the Q/P/Q circular plate.

**Figure 4:**
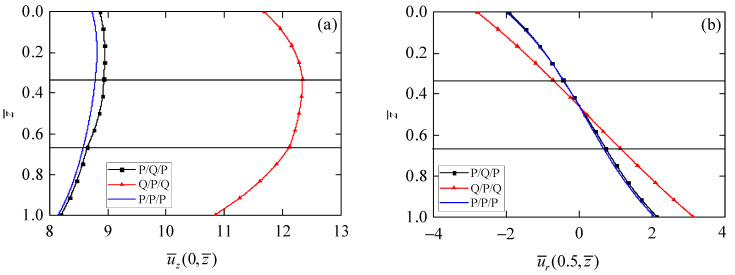

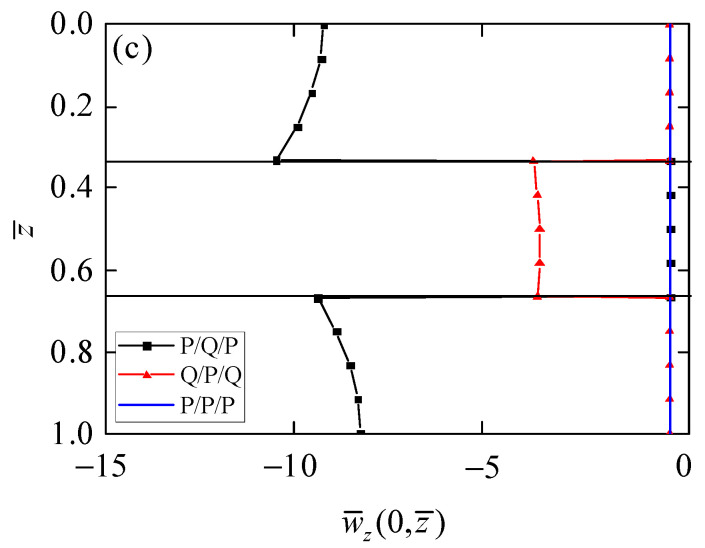
Influence of stacking sequence on phonon and phason displacement. (**a**) Phonon displacement ${\bar{u}}_{z}$; (**b**) phonon displacement ${\bar{u}}_{r}$; (**c**) phason displacement ${\bar{w}}_{z}$.

Figure 4 shows the distributions of phonon and phason displacements along the thickness of the laminated circular plates with different stacking sequences. It can be observed from Figure 4a that the axial displacement ${\bar{u}}_{z}$ is smallest in the P/P/P circular plate, while largest in the Q/P/Q circular plate. This difference provides more possibilities and selectivity for the design of laminated actuators. The radial phonon displacement ${\bar{u}}_{r}$ in Figure 4b is equal to zero at $\bar{z}$ = 0.5, which is due to the symmetry of the materials and structures; namely, the mid-plane of the laminated circular plates is the neutral plane. Furthermore, similar to the behavior of ${\bar{u}}_{z}$, the difference of ${\bar{u}}_{r}$ between the P/P/P and P/Q/P circular plates is small. However, the Q/P/Q circular plate can provide larger values of ${\bar{u}}_{r}$. Figure 4c shows that the phason displacement ${\bar{w}}_{r}$ is zero in the piezoelectric layer because there is no phason field in piezoelectric materials.

Figure 5 shows the dependences of electric potential and electric displacement on the stacking sequence of the laminated circular plates. The values of electric potential $\bar{\phi }$ (Figure 5a) for the bottom surface of circular plates with different stacking sequences are relatively close. Due to the different material composition of laminates, the values of $\bar{\phi }$ in the third layer of the P/P/P and the P/Q/P laminates increases with the decrease of the $\bar{z}$ coordinate, but $\bar{\phi }$ in the third layer of the Q/P/Q laminate decreases with the decrease of the $\bar{z}$ coordinate. It can be also found from Figure 5a that the maximum value of $\bar{\phi }$ in the Q/P/Q circular plate is smaller than that in P/P/P laminates, because the dielectric coefficient of QCs is two orders of magnitude smaller than that of piezoelectric materials. Following the same trend, we can see from Figure 5b that the maximum value of electric displacement ${\bar{D}}_{z}$ in the Q/P/Q circular plate is also smaller than that in P/P/P laminates. These phenomena of electro-mechanical coupling can provide a variety of options for the design of actuators.

## 6. Conclusions

The electro-mechanical coupling behavior of 1D piezoelectric QC laminated circular plates is studied in this paper under axisymmetric deformation conditions. Piezoelectric actuators can usually be regarded as a composite laminate structure; therefore, in order to realize the structural design of piezoelectric actuators, an accurate three-dimensional mechanical model of 1D piezoelectric QC laminated circular plates is established in the presented paper. With the aid of the state space method, finite Hankel transform, and propagator matrix, we obtain the exact axisymmetric electro-elastic solution of 1D hexagonal piezoelectric QC circular actuators under generalized elastic simply supported boundary conditions. In the numerical examples, the influences of thickness to span ratio and stacking sequence of the circular actuator subjected to top surface mechanical loading in phonon, phason, and electric fields are discussed. According to the numerical examples, we find that: (1) since no deformation assumption is introduced, the exact solution obtained can be used to verify the accuracy of the numerical results of axisymmetric bending of piezoelectric QC laminates; (2) the value of axial phonon displacement ${\bar{u}}_{z}$ in Q/P/Q circular plates for any given thickness to span ratio *s* is larger than those in the other two laminated circular plates, which reflects the better electro-mechanical coupling effect; (3) the value of phonon stress ${\bar{\sigma }}_{\theta \theta }$ is smaller when the QC layer is chosen as the outer layer of the laminated circular plates, which may help to improve the reliability of the laminated actuators; (4) although the maximum value of electric potential $\bar{\phi }$ and electric displacement ${\bar{D}}_{z}$ in Q/P/Q laminated circular plates is slightly smaller than those in P/P/P laminates, QCs have the advantage of high hardness, low thermal conductivity and so on, which can provide new design ideas for actuators working in a complex environment.

## Figures and Tables

**Table 1:** Material constants.

	1D Hexagonal Piezoelectric QCs	Piezoelectric Materials (PZT4)
Phonon elastic (Gpa)	$C_{11}=150$ $C_{12}=100$	$C_{11}=139$ $C_{12}=77.8$
	$C_{13}=90$ $C_{33}=130$	$C_{13}=74.3$ $C_{33}=115$
	$C_{44}=50$	$C_{44}=25.6$
Phason elastic (Gpa)	$\begin{array}{l} K_{1}=0.18\;\;\\ K_{2}=0.30 \end{array}$	
Coupling (Gpa)	$\begin{array}{l} R_{1}=-1.50\;\;\\ R_{2}=1.20\;\;\\ R_{3}=1.20\; \end{array}$	
Piezoelectric (C/m^2^)	$e_{31}=-0.160$	
	$e_{33}=0.347$	$e_{31}=-5.2$
	$e_{15}=-0.138$	$e_{33}=15.1$
	$d_{15}=-0.160$	$e_{15}=12.7$
	$d_{33}=0.350$	
Dielectric (C^2^·N^−1^ m^−2^)	$\xi_{11}=82.6\times 10^{-12}$	$\xi_{11}=6.46\times 10^{-9}$
	$\xi_{33}=90.3\times 10^{-12}$	$\xi_{33}=5.62\times 10^{-9}$

**Table 2:** Comparison of *z*-direction phonon displacements.

*s*	P/Q/P	Q/P/Q	P/P/P
0.1	4249.292	6688.337	4194.802
0.2	275.8201	426.0097	272.1027
0.3	57.82975	86.74357	56.99289
0.4	19.7639	28.57065	19.45356
0.5	8.855762	12.27743	8.704454
0.6	4.710162	6.246838	4.622687
0.7	2.815862	3.569921	2.759141
0.8	1.829975	2.218651	1.790067
0.9	1.264742	1.467806	1.234922
1.0	0.915595	1.018318	0.892282

## Data Availability

Not applicable.

## Appendix A

$$\begin{array}{l} \alpha_{1}=1/{\bar{C}}_{44};\;\;\;\;\;\;\alpha_{2}=s{\bar{R}}_{3}/{\bar{C}}_{44};\;\;\;\;\;\;\alpha_{3}=s{\bar{e}}_{15}/{\bar{C}}_{44};\;\;\;\;\;\;\alpha_{4}=s^{2}\left(-{\bar{C}}_{44}{\bar{K}}_{2}+{\bar{R}}_{3}^{2}\right)/{\bar{C}}_{44};\\ \alpha_{5}=s^{2}\left(-{\bar{C}}_{44}{\bar{d}}_{15}+{\bar{e}}_{15}{\bar{R}}_{3}\right)/{\bar{C}}_{44};\;\;\;\;\;\alpha_{6}=s^{2}\left({\bar{e}}_{15}^{2}+{\bar{C}}_{44}{\bar{\xi }}_{11}\right)/{\bar{C}}_{44};\\ \beta_{1}=(-{\bar{C}}_{33}{\bar{e}}_{31}^{2}{\bar{K}}_{1}+2{\bar{C}}_{33}{\bar{d}}_{33}{\bar{e}}_{31}{\bar{R}}_{1}+{\bar{e}}_{33}^{2}{\bar{R}}_{1}^{2}-2{\bar{e}}_{31}{\bar{e}}_{33}{\bar{R}}_{1}{\bar{R}}_{2}+{\bar{e}}_{31}^{2}{\bar{R}}_{2}^{2}+{\bar{C}}_{33}{\bar{R}}_{1}^{2}{\bar{\xi }}_{33}+{\bar{C}}_{13}^{2}{\bar{d}}_{33}^{2}+{\bar{C}}_{13}^{2}{\bar{K}}_{1}{\bar{\xi }}_{33}+2{\bar{C}}_{13}{\bar{e}}_{31}{\bar{e}}_{33}{\bar{K}}_{1}\\ -2{\bar{C}}_{13}{\bar{d}}_{33}{\bar{e}}_{33}{\bar{R}}_{1}-2{\bar{C}}_{13}{\bar{d}}_{33}{\bar{e}}_{31}{\bar{R}}_{2}-2{\bar{C}}_{13}{\bar{R}}_{1}{\bar{R}}_{2}{\bar{\xi }}_{33}-{\bar{C}}_{11}{\bar{e}}_{33}^{2}{\bar{K}}_{1}-{\bar{C}}_{11}{\bar{C}}_{33}{\bar{d}}_{33}^{2}-{\bar{C}}_{11}{\bar{C}}_{33}{\bar{K}}_{1}{\bar{\xi }}_{33}+2{\bar{C}}_{11}{\bar{R}}_{2}{\bar{d}}_{33}{\bar{e}}_{33}+{\bar{C}}_{11}{\bar{R}}_{2}^{2}{\bar{\xi }}_{33})s^{2}/\beta_{0};\\ \beta_{2}=\left(-{\bar{C}}_{13}{\bar{d}}_{33}^{2}-{\bar{e}}_{31}{\bar{e}}_{33}{\bar{K}}_{1}+{\bar{d}}_{33}{\bar{e}}_{33}{\bar{R}}_{1}+{\bar{d}}_{33}{\bar{e}}_{31}{\bar{R}}_{2}-{\bar{C}}_{13}{\bar{K}}_{1}{\bar{\xi }}_{33}+{\bar{R}}_{1}{\bar{R}}_{2}{\bar{\xi }}_{33}\right)s/\beta_{0};\\ \beta_{3}=\left({\bar{C}}_{13}{\bar{d}}_{33}{\bar{e}}_{33}-{\bar{e}}_{33}^{2}{\bar{R}}_{1}+{\bar{e}}_{31}{\bar{e}}_{33}{\bar{R}}_{2}+{\bar{C}}_{13}{\bar{R}}_{2}{\bar{\xi }}_{33}-{\bar{C}}_{33}{\bar{d}}_{33}{\bar{e}}_{31}-{\bar{C}}_{33}{\bar{R}}_{1}{\bar{\xi }}_{33}\right)s/\beta_{0};\\ \beta_{4}=\left({\bar{C}}_{33}{\bar{e}}_{31}{\bar{K}}_{1}-{\bar{C}}_{13}{\bar{e}}_{33}{\bar{K}}_{1}-{\bar{C}}_{33}{\bar{d}}_{33}{\bar{R}}_{1}+{\bar{C}}_{13}{\bar{d}}_{33}{\bar{R}}_{2}+{\bar{e}}_{33}{\bar{R}}_{1}{\bar{R}}_{2}-{\bar{e}}_{31}{\bar{R}}_{2}^{2}\right)s/\beta_{0};\\ \beta_{5}=\left({\bar{d}}_{33}^{2}+{\bar{K}}_{1}{\bar{\xi }}_{33}\right)/\beta_{0};\;\;\;\;\;\;\beta_{6}=-\left({\bar{d}}_{33}{\bar{e}}_{33}+{\bar{R}}_{2}{\bar{\xi }}_{33}\right)/\beta_{0};\;\;\;\;\;\;\beta_{7}=\left({\bar{e}}_{33}{\bar{K}}_{1}-{\bar{d}}_{33}{\bar{R}}_{2}\right)/\beta_{0};\;\\ \beta_{8}=\left({\bar{e}}_{33}^{2}+{\bar{C}}_{33}{\bar{\xi }}_{33}\right)/\beta_{0};\;\;\;\;\;\;\beta_{9}=\left({\bar{C}}_{33}{\bar{d}}_{33}-{\bar{e}}_{33}{\bar{R}}_{2}\right)/\beta_{0};\;\;\;\;\;\;\beta_{10}=\left(-{\bar{C}}_{33}{\bar{K}}_{1}+{\bar{R}}_{2}^{2}\right)/\beta_{0};\;\\ \beta_{11}=(-{\bar{C}}_{33}{\bar{e}}_{31}^{2}{\bar{K}}_{1}+2{\bar{C}}_{33}{\bar{d}}_{33}{\bar{e}}_{31}{\bar{R}}_{1}+{\bar{e}}_{33}^{2}{\bar{R}}_{1}^{2}-2{\bar{e}}_{31}{\bar{e}}_{33}{\bar{R}}_{1}{\bar{R}}_{2}+{\bar{e}}_{31}^{2}{\bar{R}}_{2}^{2}+{\bar{C}}_{33}{\bar{R}}_{1}^{2}{\bar{\xi }}_{33}+{\bar{C}}_{13}^{2}{\bar{d}}_{33}^{2}+{\bar{C}}_{13}^{2}{\bar{K}}_{1}{\bar{\xi }}_{33}+2{\bar{C}}_{13}{\bar{e}}_{31}{\bar{e}}_{33}{\bar{K}}_{1}\\ -2{\bar{C}}_{13}{\bar{d}}_{33}{\bar{e}}_{33}{\bar{R}}_{1}-2{\bar{C}}_{13}{\bar{d}}_{33}{\bar{e}}_{31}{\bar{R}}_{2}-2{\bar{C}}_{13}{\bar{R}}_{1}{\bar{R}}_{2}{\bar{\xi }}_{33}-{\bar{C}}_{12}{\bar{e}}_{33}^{2}{\bar{K}}_{1}-{\bar{C}}_{12}{\bar{C}}_{33}{\bar{d}}_{33}^{2}-{\bar{C}}_{12}{\bar{C}}_{33}{\bar{K}}_{1}{\bar{\xi }}_{33}+2{\bar{C}}_{12}{\bar{R}}_{2}{\bar{d}}_{33}{\bar{e}}_{33}+{\bar{C}}_{12}{\bar{R}}_{2}^{2}{\bar{\xi }}_{33})s^{2}/\beta_{0}.\;\;\; \end{array}$$
